# The Role of Inflammation in Lymphedema: A Narrative Review of Pathogenesis and Opportunities for Therapeutic Intervention

**DOI:** 10.3390/ijms25073907

**Published:** 2024-03-31

**Authors:** Catharine Bowman, Stanley G. Rockson

**Affiliations:** 1 Division of Cardiovascular Medicine, Stanford University School of Medicine, Stanford, CA 94305, USA; bowmanc@stanford.edu; 2 Department of Epidemiology and Population Health, Stanford University School of Medicine, Stanford, CA 94305, USA

**Keywords:** lymphedema, inflammation, lymphatic system, pathogenesis, therapeutic, review, lymphatic disease, lymphoedema

## Abstract

Lymphedema is a chronic and progressive disease of the lymphatic system characterized by inflammation, increased adipose deposition, and tissue fibrosis. Despite early hypotheses identifying lymphedema as a disease of mechanical lymphatic disruption alone, the progressive inflammatory nature underlying this condition is now well-established. In this review, we provide an overview of the various inflammatory mechanisms that characterize lymphedema development and progression. These mechanisms contribute to the acute and chronic phases of lymphedema, which manifest clinically as inflammation, fibrosis, and adiposity. Furthermore, we highlight the interplay between current therapeutic modalities and the underlying inflammatory microenvironment, as well as opportunities for future therapeutic development.

## 1. Introduction

The lymphatic system plays a critical role in fluid homeostasis, immunity, and lipid transportation [1,2,3,4]. In the presence of lymphatic injury or a predisposition to lymphatic dysfunction, these processes are disrupted, leading to an interruption of lymphatic function [5]. Lymphedema is an incurable disease of the lymphatic system that results from an initial lymphatic injury/dysfunction and the subsequent activation of a pro-inflammatory cascade, placing the lymphatic system in a state of chronic inflammation. Despite its widespread impact, our understanding of the role of inflammation in mediating lymphedema is still rapidly evolving [6]. Furthermore, the ability to harness this inflammatory microenvironment is of great interest to targeted therapeutic interventions [7,8]. Therefore, the aim of this review is to provide an overview of the known inflammatory mechanisms underlying lymphedema development and pathogenesis, as well as highlight areas of current and anticipated therapeutic intervention for its treatment. We will discuss lymphatic physiology and pathophysiology, followed by the unique mechanisms of acute and chronic lymphedema development. Finally, we will review current treatments and areas for future therapeutic intervention.

## 2. The Lymphatic System

The lymphatic system is a complex physiological network of lymphoid organs and vessels that parallel the cardiovascular system [5,9]. The lymphatic vasculature is designed to facilitate the transport of lymph, a protein- and immune cell-enriched fluid, between the interstitial and intravascular compartments [1,10]. Much of our earliest understanding of this physiological system stems from its role in maintaining fluid homeostasis. Lymphatic capillaries are lined with glycosylated intercellular junctions that facilitate fluid transport across vascular borders, according to Starling’s Law [11,12]. Fluid transit into the interstitium is, therefore, a physiologic function of both hydraulic and oncotic pressures, as well as the cross-capillary membrane protein gradient generated by lymph/interstitial fluid [12,13]. Upon uptake, lymph is then propelled to larger, more robust pre-collecting vessels and finally enters the minimally permeable network of lymphatic collectors [1,14]. These lymphatic collectors act as the mechanical pump of the lymphatic system, encased and surrounded by smooth muscle that pushes fluid through the system by peristaltic contraction. Retrograde flow of lymph is prevented due to the presence of lymphatic chambers (lymphangions) that are defined by the presence of proximal and distal unidirectional lymphatic valves [10,11]. Intra-chamber pressures and local chemical mediators interact with the lymphatic endothelium and surrounding smooth muscle cells to promote peristalsis [15,16,17]. Throughout its journey through the lymphatic vasculature, lymph transports cellular debris, proteins, microorganisms, and cells from the interstitium and delivers them to lymph node basins for filtration, clearance, and/or immune recognition prior to the return of the lymph into the venous circulation at the lymphovenous junction with the subclavian vein [1,2].

The role of the lymphatic system in mediating immune function is critical. At the level of the lymph node, lymph traverses the subcapsular sinus, guided by a chemical gradient of C-C Motif Chemokine Ligand 21 (CCL21) that facilitates mobilization, conditioning and antigen presentation of immune cells (predominantly dendritic cells [DCs] and memory T cells) [18,19]. Pro-inflammatory cytokines and molecules allow for lymph node remodeling during the inflammatory response, encouraging resident T-cell accumulation. Antigen-presenting cells or small free antigens therefore localize to resident T-cell zone within the lymph node for proper interaction and processing by DCs [9,20]. Larger molecules are unable to traverse the small passages within the lymph node and therefore are processed peripherally by macrophages and B-cells. Lymphangiogenic activity then transitions from the subcapsular sinus to the medullary region of the lymph node, promoting the egress of T-cells (e.g., CD4^+^) and B-cells to regional tissues, along with sphingosine-1-phosphate release [9,21,22]. In surrounding tissue, the release of pro-inflammatory molecules promotes the mobility and activity of DCs, macrophages, neutrophils and T-helper cells. The release of soluble nitric oxide (NO) via endogenous NO synthase (eNOS) and vascular endothelial growth factor-c (VEGF-C) from lymphatic endothelial cells, B-cells, and macrophages, facilitates the necessary changes in lymphatic architecture and contractility to encourage the movement of lymph throughout the body [23,24]. In the presence of lymphatic pathologies, however, VEGF-C and NO production via inducible NOS (iNOS) potentiates lymphatic damage [25].

The third role of the lymphatic system is in the regulation of lipid transport and adiposity. Intestinal lacteals are known for their uptake of chylomicrons and transformation of free fatty acids during digestion [26,27]. These absorptive lymphatic vessels undergo constant postnatal restructuring and lymphangiogenesis, regulated by VEGF-C [28]. Interestingly, peripheral lymphatic vessels have been more recently identified to be capable of facilitating the transport of larger cholesterol molecules, previously thought to reside only within the blood vasculature [26,29,30,31,32]. The lymphatic system balances the many roles it must play in maintaining bodily function. Hence, when lymphatic injury or predisposition to lymphatic dysfunction are present, the intricate balance of these functions is offset, resulting in complex, systemic lymphatic disease.

## 3. Lymphedema: An Overview

### 3.1. Definition and Epidemiology

Lymphedema is a chronic disease of the lymphatic system characterized by impaired lymphatic drainage, local immune dysfunction, adipose deposition and chronic inflammation. Globally, it is estimated that up to 250 million individuals are affected by lymphedema [33]. Lymphedema can be primary or secondary [34]. Primary lymphedema is caused by a constitutional predisposition to lymphatic dysfunction, occurring as the primary disease alone or coupled with other syndromic features. Primary lymphedema has an estimated prevalence of 0.1% [35]. Lymphedema may alternatively occur as a result of a serious injury, systemic stressor, infection, surgery, or obstruction to the lymphatic system. This form of lymphedema, commonly referred to as secondary or acquired lymphedema, is considered neither rare nor common [35]. In North America, cancer-related treatments, such as radiation and lymphadenectomy, are among the leading causes of secondary lymphedema, with up to 69% of patients developing lymphedema after cancer therapy [36,37]. Other conditions, such as obesity, are becoming increasingly recognized as common systemic stressors that lead to secondary lymphedema [38,39,40]. Furthermore, infections, such as the parasite Wuchereria Bancrofti, can lead to secondary filarial lymphedema [41].

Regardless of origin, it is well-documented that lymphedema has a significant, negative impact on quality of life and psychosocial well-being, globally [42,43,44,45,46,47]. As a consequence of local immune dysfunction, lymphedema patients are subject to recurrent soft tissue infections that can lead to repeated hospitalization. These infections are potentially life-threatening if left untreated [4,48,49,50,51,52]. Furthermore, as a result of swelling and adipose tissue expansion in later-stage disease, patients may experience psychosocial distress related to physical appearance and loss of function [47]. Patients also face stigmatization that takes unique forms depending upon cultural and regional contexts. For instance, a study conducted in the Dominican Republic and Ghana demonstrated that women with filarial lymphedema face extreme stigmatization relating to appearance, perceived care burden, and fear of contagion. This stigmatization and associated negative self-perception of appearance have also been documented in other global regions including India, Nigeria, and the United States [53,54,55,56]. These psychosocial experiences interact with the physical characteristics of lymphedema to create a complex network of experiences that implicate patient well-being, regardless of lymphedema sub-type or global region. The global impact of lymphedema further extends to cost burden. Several studies have been undertaken that demonstrate the significant healthcare costs endured as a result of lymphedema, which is regionally distributed. In France, for instance, it has been shown that annual out-of-pocket care costs are six times that of the average outpatient care costs for the general population with total out-of-pocket costs representing up to 10.1% of income per consumption unit [57]. In India, where community-based care interventions have been introduced for the treatment of filarial lymphedema, individual patient savings were 185 times the cost of the program’s individual costs [58].

### 3.2. Clinical Characterization

Lymphedema is characterized by regional lymphatic dysfunction and the eventual accumulation of protein-rich interstitial fluid, followed by increased adipose deposition due to the presence of free fatty acids in accumulated lymph. The International Society of Lymphology has identified a four-stage grading system for lymphedema to guide clinical diagnosis and treatment [59]. Stage 0 is considered pre-clinical disease without edema in the affected region despite the presence of impaired lymphatic drainage, as indicated by lymphatic imaging. Stage I lymphedema is characterized by dynamic interstitial fluid accumulation that is relieved through elevation. Stage II lymphedema occurs with dermal fibrosis and worsening fluid accumulation that is not readily reducible by elevation or compression. Stage III is the most advanced stage of disease, with irreversible non-pitting edema, and marked cutaneous and subcutaneous tissue deformation, including increased adiposity, fibrosis, and keratinic deposits.

## 4. Lymphedema: An Inflammatory Pathophysiologic Process

### 4.1. The Initial Phases of Lymphedema

**Figure 1:**
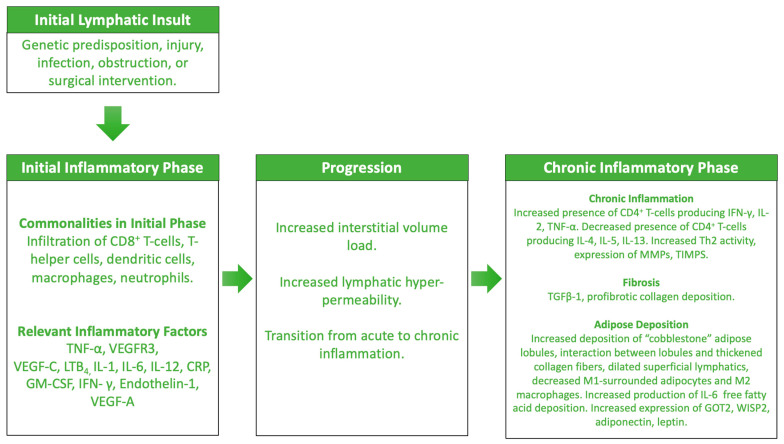
Overview of the mechanisms known to contribute to the development and pathogenesis of lymphedema.

It was previously thought that lymphedema occurred as a direct response to local lymphatic injury or mechanical dysfunction. However, it has become apparent that this mechanical disruption is only part of the broader story that explains the pathogenesis (Figure 1). In the initial phases of disease, the lymphatic system endures the first of “two hits” against its function: a genetic predisposition, injury, infection, obstruction, or surgical intervention impairs lymphatic function, tipping the balance away from systemic homeostasis [60,61]. With an increased flux of interstitial fluid, the capacity of the system to accommodate such local flow and pressure fails, releasing a cascade of ensuing inflammatory events that establishes long-term disease and irreversible tissue transformation [1,61,62]. The initial inflammatory response associated with lymphatic damage is dependent upon the nature of the initial assault [5,41,61,63,64,65,66]. There are, of course, certain commonalities within the overarching inflammatory cascades that are activated, including the involvement of CD8^+^ T-cells, T-helper cells, macrophages, and neutrophils, and certain pro-inflammatory cytokines, such as tumor necrosis factor alpha (TNF)-α, VEGF-C, and leukotriene-B_4_ (LTB_4_), all of which promote acute and chronic inflammatory processes [64,66,67,68,69,70].

In the case of primary lymphedema, several genes have been identified that are associated with the dysfunction of valvular structures or inter-cellular junctions, and with hyper- and hypotrophic vascular changes, all of which contribute to the overt initial lymphatic injury [71,72,73,74,75,76,77,78,79,80,81,82,83]. Given the nature of primary lymphedema as a rare condition in which we have scant methodology to establish the onset of an inflammatory assault, few studies have investigated the underlying inflammatory changes that accompany the first disease presentation. This is not the case, however, for forms of secondary lymphedema, where an initial lymphatic insult is quite apparent. In the case of secondary filarial lymphedema, both innate and adaptive immune responses are activated [63,64,84,85,86], where innate immunity participates in the immediate response to intravascular invasion by Wuchereria Bancroffti [41]. Early studies of murine models lacking either T-cells alone or with combined T- and B-cell deficiency demonstrated acute lymphangitis [87,88]. However, after reconstitution with splenic immune cells, a more progressive inflammatory transformation occurred, characterized by fibrosis, lymphatic thrombi, and increased lymphangitis, suggesting adaptive immune involvement. On the molecular level, later studies demonstrated that T-cell-deficient mice with Brugaria infection exhibited higher levels of pro-inflammatory cytokine expression in lymph, including Interleukin (IL)-1, IL-6, TNF-α and granulocyte-macrophage colony-stimulating factor (GM-CSF) [87,89,90]. These findings have been echoed in studies of human lymphatic filariasis, where elevated levels of TNF-α, TNF-receptor, IL-6, IL-2, interferon-γ (IFN-γ), endothelin-1, and C-reactive protein have been identified [91,92,93,94]. Few directed analyses have been undertaken of lymph fluid derived from patients with lymphatic filariasis; however, one study did report increased levels of IL-1β in circulating lymph. In addition to innate immune modulation, studies of overt filarial infection (i.e., without manifestations of clinical lymphedema) have demonstrated reduced CD4^+^ T-cell activation as a result of increased dendritic cell (DC) death [95]. However, the augmented presence of activated CD8^+^ T-cells has since been identified in circulating blood, skin and subcutaneous samples [96,97].

It should not be surprising that other forms of secondary lymphedema, such as post-operative or injury-acquired lymphedema, are also accompanied by a unique inflammatory fingerprint. Direct comparison of filarial and post-operative cutaneous tissues reveals less involvement of CD68^+^, CD4^+^ and CD8^+^ T-cells within non-filarial lymphedematous skin tissue [97,98,99]. This finding supports the hypothesis that the initial inflammatory insult that occurs in lymphatic filariasis has a dual impact, with both mechanical and infectious components that are able to elicit an immune response. Despite the lesser prevalence of CD4^+^ T-cells in post-operative cutaneous samples, a defining feature of the initial inflammatory response in this form of lymphedema includes infiltration of CD4^+^ T-cells, CD8^+^ T-cells, neutrophils, DCs and macrophages [95,100,101,102]. Interpretation of the role of macrophages in lymphedema pathogenesis is complicated by the dual macrophage phenotype (M1 and M2) [103]. M1 is the pro-inflammatory phenotype of the macrophage, associated with further tissue damage and inflammatory changes to the regional microenvironment. In contrast, M2 is commonly referred to as a regenerative macrophage phenotype, releasing factors such as VEGF-A and C to support lymphangiogensis and local lymphatic tissue transformation. Other molecules, such as IL-10, transforming growth factor β (TGF-β), LTB_4_ and extracellular matrix proteins, are also produced by M2 macrophages [52,67,103,104]. In lower concentrations, LTB_4_, a downstream product of 5-lipoxygenase (5-LO) enzymatic activity, can elicit prolymphangiogenic effects, whereas higher concentrations impede both VEGFR3 and Notch signaling [66,67]. LTB_4_ has been further recognized for its ability to promote CD4^+^ and CD8^+^ cell recruitment and Th17 cell differentiation within lymphedematous tissues [105]. M2 macrophages can further facilitate decreased IL-12 expression, perhaps owing to its association with Th2-mediated immunity [106]. There is a robust body of ongoing investigations that describe the role of T-helper cells in the inflammatory pathogenesis of lymphedema. For instance, Th2 cells have been shown to produce TGF-β, which leads to downstream tissue fibrosis and, furthermore, increases the expression of IL-3 and IL-4 within local tissues, leading to lymphangiogenic changes through direct suppression of the lymphatic endothelium [107]. Other T-helper cell populations, including Th1 and Th17 cells, are localized within regions of fibrosis and lymphedema [108,109].

### 4.2. The Prolonged Phases of Lymphedema

Over time, these processes contribute to an overall state of chronic inflammation local to and beyond the original site of lymphatic assault (Figure 1). As the shift towards a preferential adaptive immune response occurs, additional changes in lymphatic tissues are observed, resulting in the final defining features of progressive, severe lymphedema, namely, tissue fibrosis and increased adiposity [110]. In long-standing filarial lymphedema, there is an increased population of CD4^+^ cells producing IFN-γ, IL-2 or TNF-α, while CD4^+^ T-cells expressing IL-4, -5, or -13 are markedly decreased compared to asymptomatic patients. Matrix metalloproteinases (MMPs) and associated inhibitors (tissue inhibitors of MPs, [TIMPS]) are also increasingly expressed by regional immune cells, including macrophages, granulocytes, epidermal cells, and fibroblasts [64]. It is hypothesized that an imbalance between MMP and TIMP expression leads to the fibrotic phenotype during filarial lymphedema. Other cytokines more generally associated with fibrogenesis (e.g., IL-4, IL-5, IL-13, and TGF-β1 ) are also expressed in local tissues. The upregulation of Th2 activity across the various causes of sustained lymphedema is well-established, thereby leading to the increased production of profibrotic cytokines [64,86,111]. In lymphedematous murine models, however, resolution of fibrosis has been demonstrated through the introduction of neutralizing IL-4 and IL-13 cytokines [87]. Late-stage depletion of macrophages has also been demonstrated in lymphedema, leading to increased accumulation of Th2 cells and increased profibrotic collagen deposition. Sustained inflammation within the lymphedematous region leads to the degradation both of lymphatic structures, leading to further impairment of lymphatic vascular function, and of the surrounding tissues. The state of chronic inflammation sustains a vicious feedback loop of pro-inflammatory cytokine expression, fibrosis and excess adipose tissue deposition.

Increased adipose tissue deposition is a well-characterized finding in human and animal models of sustained lymphedema. On ultrasound, adipose tissue lobules appear as a “cobblestone” pattern [112]. Interactions between lobular adipose structures and thickened collagen fibers suggest a linkage between fibrogenecity and the adiposity associated with prolonged lymphatic dysfunction. Dilated superficial lymphatic vessels have also been found within lymphedematous adipose tissue, providing a potential link between worsening lymphatic flow and adipogenesis. Furthermore, a decreased presence of M1-surrounded adipocytes and M2 macrophages were noted within lymphedematous adipose tissue when compared to non-lymphedematous controls [112,113]. The excess adiposity associated with lymphedema is thought to occur as a result of the expression of specific inflammatory cytokines, such as IL-6, and increased free fatty acid deposition in lymphedematous tissues attributable to the sustained impairment of lymph flow. An increased expression of regulators of adipogenesis, glutamic-oxaloacetic transaminase-2 (GOT2) and WISP2, has been identified in lymphedema-associated adipose tissue [111]. Interestingly, the correlation of expression profiles of perilipin (PLIN)-1 and -3 and podoplanin (PDPN) has been hypothesized as a potential link between lymphangiogenesis and lipolysis, but has not yet been explored in the context of lymphedema. Patients with lymphedema also express increased adiponectin and leptin in serum samples (and a reduced adiponectin/leptin ratio) [111,114], outlining the potential hormonal pathway through which increased adiposity manifests within lymphatic patients. Hence, although macroscopic identification of adipose deposition is becoming better-established in chronic lymphedema, there remains much to be understood regarding the molecular mechanisms regulating this aspect of lymphedema pathogenesis.

## 5. Lymphedema: Current Therapies

### 5.1. The Relationship between Treatment and Inflammation

As discussed, there is a growing list of clues to the molecular mechanisms that govern the pathogenesis of lymphedema. These insights are critical to the development of new, targeted lymphedema therapeutics. As a reflection of the earliest conception of lymphedema as a form of pure mechanical vascular disruption, existing lymphedema therapies are inherently mechanical in nature. However, research is beginning to reveal that, even through mechanical manipulation, much of the inflammatory microenvironment can be modified (Table 1).

### 5.2. Complete Decongestive Therapy

The current therapeutic gold standard for lymphedema is represented by a collection of manual physical therapies that aim to mobilize accumulated interstitial fluid from the affected regions back into the blood vascular circulation [128]. The compression and massage components of complete decongestive therapy (CDT) attempt to promote regional contractility and thereby optimize lymphatic vascular function [129,130]. In recent years, studies have shown that not only can CDT reduce accumulated fluid volume, but the act of encouraging lymph transport back to the central blood vascular circulation may also lead to anti-inflammatory changes at a molecular level. Recent studies have demonstrated that circulating levels of TNF-α, IL-10 and monocytes in lymphedema patients was significantly reduced following CDT when compared to control participants [115]. Other studies have investigated local hyaluronic acid clearance after CDT; the expression of this well-recognized fibrogenic factor is enhanced eightfold in lymphedematous tissues when compared to controls [131]. Although hyaluronic acid levels did not differ before and after a 3-week course of CDT, aldosterone was significantly increased [116]. Similar studies were undertaken in patients with head and neck lymphedema after 8 weeks of pneumatic compression pump use. In this randomized control trial of 43 patients, no significant differences were identified in the blood levels of IFN-γ, TNF-α, TGFβ-1, IL-1β, and IL-6, despite symptomatic improvement [117]. Collectively, these studies suggest a potential impact of manual physical therapies on the underlying inflammatory microenvironment associated with lymphedema. Further research is needed to better elucidate this relationship in terms of correlation with treatment outcomes, length, and intensity.

### 5.3. Surgical Intervention

Surgical intervention for lymphedema is another treatment modality that is becoming increasingly available to patients. Surgery often falls within two primary categories: (i) physiologic and (ii) reductive techniques [132,133]. Physiologic techniques aim to provide an enhancement of lymph flow within regions of damaged lymphatic vasculature. Most often, these physiologic procedures take the form of either vascularized lymph node transfer or lymphaticovenous anastomosis. With vascular lymph node transfer, nodal structures and surrounding vasculature are harvested from an unaffected region of the body and transplanted locally to the area of lymphedematous involvement. It is hypothesized that the engrafted vascularized node transplant encourages local lymphatic regeneration. An analysis of post-operative wound exudates was undertaken to compare inflammatory cytokine profiles in patients with axillary lymph node dissection, breast reconstruction, microvascular lymph node transfer and a combined reconstruction-transplant approach [118]. These studies found that patients with the combined procedure had the highest production of the anti-inflammatory and antifibrotic cytokine, IL-10. Differences in the pro-lymphangiogenic growth factor, VEGF-C were also found among the lymph node dissection group and reconstruction/combined transfer-reconstruction groups [119]. Separate studies have corroborated these findings, noting correlation between IL-10, TNF-α, and TGFβ-1 levels and lymphedema-related factors following lymph node transfer [118]. In the context of lymphaticovenous anastomosis, histological analyses of skin samples showed decreased CD4^+^ cell inflammation in the lymphedematous limb biopsies, whereas the control limbs showed no difference [120]. Changes were further associated with decreased collagen type I deposition and TGFβ-1 expression, implicating antifibrotic activity six months post-lymphaticovenous anastomosis [120]. More recent work has focused specifically on characterizing the peripheral T-cell profile in lymphedema patients after lymphaticovenous anastomosis [121]. A one-year pre-post comparative study of lymphedematous tissue exhaustion and inflammation demonstrated that IFN-γ and IL-17A levels in CD4^+^ cells were downregulated following lymphaticovenous anastomosis, while T-cell receptor diversity increased post-operatively, a measure that is typically low in lymphedematous tissues. Hence, it is suggested that the inflammatory profile can be improved using physiologic techniques; however, additional work in this area should be conducted to verify and further characterize these outcomes. Reductive surgical techniques are known to intervene upon the later stages of lymphedema pathophysiology through the removal of adipose and fibrotic tissues, although fewer studies have been undertaken to analyze the potential transformation of inflammatory expression. Given the known pro-inflammatory nature of accumulated adipose tissue, the transformation of this local lymphedematous region through the removal of pro-inflammatory tissue is likely to influence the overall inflammatory microenvironment in which lymphatic structures are housed. The final form of lymphatic surgery includes preventative techniques, namely, sentinel lymph node biopsy, axillary reverse mapping, and lymphedema microsurgical preventive healing approach (LYMPHA) [134]. These procedures aim at surgical vascular anastomosis following the active surgical removal of cancer. Although this form of treatment may not address the many complexities that in the face of clinically overt lymphedema, LYMPHA may provide patients with an opportunity to decrease lymphedema risk and, ultimately, disease burden, averting or minimizing lymphedema onset.

## 6. Lymphedema: Future Therapies and Challenges in Development

### 6.1. Targetting Inflammation for Future Therapeutics

Given the invasive nature of surgical interventions for lymphedema, and the incomplete nature of the ongoing research into their long-term efficacy, alternative approaches to innovative lymphedema therapies are being explored. Many of these therapies are focused upon identifying pharmacologic interventions that target the inflammatory microenvironment that characterizes this disease (Table 1). Over the last two decades, these therapies have ranged from benzopyrones to immunosuppressant medications, such as tacrolimus [135,136]. Despite their differing mechanisms of action and the limitations of the associated clinical studies, most pharmacological solutions that have been proposed for lymphedema to date have targeted the underlying inflammatory mechanisms discussed in this review. Below, we have highlighted a few areas in which substantial progress has been made.

### 6.2. 5-Lipoxygenase Targeting Medications

There have been multiple studies investigating the utility of a non-steroidal anti-inflammatory (NSAID) drug (ketoprofen) as a treatment for lymphedema. In earlier murine models of lymphedema, modulation of the 5-lipoxygenase pathway was found to mitigate fluid accumulation and pro-inflammatory histological tissue transformation correlated with impaired immune cell recruitment [67]. These early studies provided a potential mechanism through which the inflammation and, potentially, longer-term manifestations of lymphedema (i.e., fibrosis, adiposity) could be targeted for therapeutic benefit. Hence, ketoprofen, an NSAID known to uniquely antagonize the 5-lipoxygenase pathway, was systemically administered in a murine model of lymphedema, demonstrating therapeutic benefit and was, therefore, trialed in humans [122,123]. While ketoprofen has the demonstratable ability to decrease dermal thickness and G-CSF in patients, paralleling the murine observations, a black box warning against the prolonged use of NSAIDs was subsequently issued, highlighting the risks of cardiovascular toxicity [137], dampening excitement for the use of ketoprofen in lymphedema. Results from these initial studies on ketoprofen, however, provided important insights into more relevant targets within the 5-lipoxygenase axis. Again investigating the murine model, the authors noted that the targeted antagonism of LTB_4_, a downstream mediator, provides comparable therapeutic benefits, with demonstrable enhancement of the molecular machinery that subserves lymphatic regeneration, and without the attendant cardiovascular risks [122]. The first trial investigating direct LTB_4_ antagonism (the ULTRA trial) used a medication called Ubenimex [6]. The ULTRA Trial was terminated prematurely based upon findings; with insufficient power to assess the primary endpoint, the aborted trial was inconclusive (unpublished observations). However, a more conclusive Phase II trial, using Acebilustat, a highly specific LTA_4_ hydrolase antagonist, is now underway [138].

### 6.3. Antifibrotic Medications

Fibrosis is a well-recognized attribute of lymphedema, implicating tissue integrity and disease control. One of the primary factors involved in lymphedema-associated fibrogenesis is TGFβ-1, rendering this molecule a logical target for the prevention of lymphedema progression. Anti-TGFβ-1 treatments have thus far only been introduced in the pre-clinical context. The use of neutralizing anti-TGF antibodies has been explored in the context of animal models of lymphedema, demonstrating increased collateral lymphatic formation and the inhibition of T-cell infiltration [107]. Furthermore, EW-7197, a peroral TGF-β type I receptor kinase inhibitor, has a demonstrable favorable impact on fibrosis, interstitial flow and lymphangiogensis within murine models of tail lymphedema [124]. Ultimately, the targeting of fibrosis for the treatment of lymphedema is an evolving area of exploration, with research still in the pre-clinical phase. This presents an opportunity for further investigation and, ideally, the expansion of pre-clinical studies into translational phases of work in the future.

### 6.4. Tacrolimus

The final area of discussion on future pharmacological solutions for lymphedema involves tacrolimus, an anti-T-cell agent that is FDA-approved for its use in the topical therapy of skin inflammation and fibrotic diseases [125]. In murine models of lymphedema, topical tacrolimus exhibited improved lymphatic contractility, swelling, T-cell infiltration, and tissue fibrosis, while also facilitating the formation of lymphatic collateral vessels and decreasing backflow [125,126,127]. Ultimately, this is an intriguing area for additional research, however, more robust scientific evidence must be generated prior to introducing tacrolimus within human trials.

### 6.5. Challenges for Therapy Development and Translation

One of the core challenges associated with translation, particularly in capturing the longitudinal course of lymphedematous tissue transformation, is the spontaneous resolution of lymphedema that occurs in murine models of lymphedema, including the commonly used mouse tail model. Hence, Jørgensen and colleagues [139] explored eight revised approaches to the murine lymphedema tail model to address this limitation. Jørgensen and colleagues [139] found that one model, which used surgical lymphatic ablation with two fractions of 10-Gy irradiation, induced the impairment of lymphatic drainage, lymphatic ectasis, and increased limb volumes that was maintained over eight weeks without adverse events. Other limitations, such as the relative size of the lymphedematous region and differing gravitational influences on lymph accumulation have also been taken into consideration as limitations of current pre-clinical models. The use of Yucatan minipigs has been proposed as a larger-scale model of lymphedema that better accounts for these limitations [140,141]. However, this species lacks the critical axillary lymph node architecture needed to address predominant forms of axillary lymphadenectomy-induced lymphedema. Ultimately, comprehensive representation of the many nuances associated with human lymphedema in one singular pre-clinical model and/or inter-species comparability continue to create significant challenges for effective translation.

## 7. Conclusions

The underlying inflammatory mechanisms of lymphedema have been an area of substantial investigation and serve as an important area of research for the prevention and treatment of this chronic, progressive disease. In future iterations of this work, a systematic review approach would add additional value to ensure the full breadth of this literature is captured. The current review addresses the inflammatory pathogenesis underlying the acute and sustained aspects of lymphedema: inflammation, fibrosis, and adipose tissue deposition. Interventions acting upon earlier phases of lymphedema appear to not only provide symptomatic relief, but also modulate the inflammatory microenvironment that, unaddressed, lead to lymphatic disease progression. Further studies are needed to strengthen therapeutic defenses against disease progression.

## Figures and Tables

**Table 1:** The impact of current and prospective lymphedema treatments on underlying inflammatory mechanisms that promote lymphedema development and progression.

**Current Therapies**		
**Treatment**	**Description**	**Reported Impact on Inflammatory Mechanisms**
Complete Decongestive Therapy	Manual physical therapies (e.g., compression, massage) that aim to mobilize accumulated interstitial fluid from affected regions back into blood vascular circulation.	Complete Decongestive Therapy (CDT) Decreased circulating levels of TNF-α, IL-10, monocytes [115]. Increased aldosterone, no significant change in hyaluronic acid levels after 3-weeks of CDT [116]. Pneumatic Compression No significant difference (head and neck) in blood levels of IFN-γ, TNF-α, TGFβ-1, IL-1β , IL-6 after 8-weeks of pneumatic compression [117].
Surgical Interventions	Physiologic and reductive techniques, including lymphaticovenous anastomosis, vascularized lymph node transfer, breast reconstruction, combined approaches.	Lymph Node Transfer and Combined Techniques Increased production of IL-10 (after combined lymph node transfer and anastomosis) [118]. Modulation of VEGF-C production, correlation between IL-10, TNF-α, TGFβ-1 and lymphedema-related factors following lymph node transfer [119]. Lymphaticovenous anastomosis Decreased CD4^+^ cell inflammation, hyperkeratosis, epidermal proliferation, collagen type I deposition and TGFβ-1 expression (biopsy) [120]. One-year post-operative decrease in IFN-γ and IL-17A expression, increased T-cell receptor diversity. Downregulation of PD-1, Tim-3, PD-1^+^Tim-3^+^ on CD4^+^ and CD8^+^ T cells [121].
**Novel Therapies**		
**Treatment**	**Description**	**Reported Impact on Inflammatory Mechanisms**
5-Lipoxgenase Targeting Medications	Ketoprofen, bestatin (Ubenimex), and Acebilustat are known modifiers of the 5-lipoxygenase pathway that leads to lymphedema progression and worsening.	Ketoprofen Decreased dermal thickness, improved histopathological scores (dermal thickness, collagen thickness, intercellular mucin deposits, perivascular inflammation), decreased plasma G-CSF (human) [122]. Upregulation of VEGF-C, VEGFR-3, PROX-1 expression and paradoxical increase in TNF-α. Normalized histopathological findings of hyperkeratosis, epidermal spongiosis, edema, irregularity of epidermal/dermal junction, elongation of dermal papillae of tail (murine) [123]. Bestatin Improved lymphatic flow, decreased lymphatic permeability, diminished macrophage and neutrophil infiltration in skin sample, decreased IL-6, IL-4, IL-13, and IL-17A, elevated IL-10 (murine) [67].
Antifibrotic Medications	Anti-fibrotic medications target tissue transformation that has been found in later stages of lymphedema.	Neutralizing anti-TGF Antibodies Decreased ECM deposition, increased collateral lymphatic formation, inhibition of T-cell infiltration. Decreased tail edema, fibroadipose tissue deposition, and expression of TGFβ-1 and pSmad3 in skin, decreased expression of all TGF-β isoforms and downstream signaling molecules (Sp1, RhoA, Cfl1, Map3k7, Mapk14, RelA, Nfκb2 and Akt1) and inflammatory mediators (IL-1β, TNF-α, IL-6, -4, -13, -10, -17α) in tail tissue. Decreased skin leukocyte, CD4^+^, Th1, and Th2 cells, and neutrophils (murine) [107]. EW-7197 Improvements in fibrosis, interstitial flow, lymphangiogenesis, decreased tail diameter (murine) [124].
Tacrolimus	Tacrolimus is an anti-T-cell agent approved for topic treatment of skin inflammation and fibrosis.	Improved lymphatic contractility, swelling, T-cell infiltration, tissue fibrosis. Increased formation of lymphatic collateral vessels, decreased backflow (murine) [125,126,127].

## Data Availability

No new data were created or analyzed in this study. Data sharing is not applicable to this article.
